# Cancer cells escape p53’s tumor suppression through ablation of ZDHHC1-mediated p53 palmitoylation

**DOI:** 10.1038/s41388-021-01949-5

**Published:** 2021-07-19

**Authors:** Jun Tang, Weiyan Peng, Yixiao Feng, Xin Le, Kang Wang, Qin Xiang, Lili Li, Yan Wang, Can Xu, Junhao Mu, Ke Xu, Ping Ji, Qian Tao, Ailong Huang, Chu-Xia Deng, Yong Lin, Tingxiu Xiang

**Affiliations:** 1 Chongqing Key Laboratory of Molecular Oncology and Epigenetics, The First Affiliated Hospital of Chongqing Medical University, Chongqing, China; 2 Cancer Epigenetics Laboratory, Department of Clinical Oncology, State Key Laboratory of Translational Oncology, Sir YK Pao Center for Cancer and Li Ka Shing Institute of Health Sciences, The Chinese University of Hong Kong, Shatin, Hong Kong; 3 The Affiliated Stomatological Hospital of Chongqing Medical University, Chongqing, China; 4 MOE Key Laboratory of Molecular Biology for Infectious Diseases, Department of Infectious Disease, Chongqing Medical University, Chongqing, China; 5 Faculty of Health Sciences, University of Macau, Macau, SAR China; 6 Lovelace Respiratory Research Institute, Albuquerque, NM USA

**Keywords:** Tumour biomarkers, Post-translational modifications

## Abstract

The inactivation of tumor-suppressor genes contributes heavily to oncogenesis. The mutation of TP53 has been well-studied and recognized as a major factor in the development of tumors. Yet other means of p53 inactivation has not been well-elucidated. We previously identified a hypermethylated gene *ZDHHC1* that suppresses tumor growth when the expression was restored, but the specific mechanism was yet to be found. The protein product of *ZDHHC1* is an S-palmitoyltransferase and we have identified p53 as a substrate for ZDHHC1-mediated palmitoylation, specifically at the C135, C176, and C275 residues. The novel form of post-translational modification of p53 is required for the nuclear translocation of the tumor suppressor. p53 recruited DNMT3A to *ZDHHC1* promoter and is responsible for the hypermethylation of *ZDHHC1*. The epigenetic feedback loop formed by ZDHHC1 and p53 sheds light on the inactivation of p53 without the presence of genetic mutations.

## Introduction

Cancer development is a complex process involving oncogene activation and tumor suppressor gene inactivation by genetic and/or epigenetic mechanisms [1]. *TP53* is one of the most extensively studied tumor suppressor genes whose multifaceted mechanisms involve apoptosis, ferroptosis, DNA repair, genomic stabilization, and angiogenesis [2]. Thus, breaking through p53’s blockade is a crucial step for malignant cellular transformation. Approximately half of the human solid tumors have one or more mutations in the genetic sequence of *TP53*, which often leads to the loss of tumor-suppressing properties and even worse, the gain of tumor-promoting functions [3]. However, in cancers retaining wild-type *TP53* (*TP53*^WT^), the mechanism of tumorigenesis is not well-understood.

Beyond the genetic level, p53 activity is mainly regulated by post-translational modifications (PTMs). For example, p53 protein is activated by phosphorylation and acetylation and inactivated by ubiquitination-mediated proteasomal degradation [4]. When triggers such as DNA damage is induced, p53 is rapidly phosphorylated, which triggers its nuclear translocation to regulate the expression level of its target genes. The transcription regulating the activity of p53, either activation or repression, is further modulated by p300-mediated acetylation on the promoter [5]. However, the current understanding of p53 PTMs fails to translate into effective prevention or treatment of cancers and requires further exploration.

Protein S-palmitoylation is recently defined as another important form of post-translational modification in tumorigenesis [6, 7], which is catalyzed by polytopic transmembrane proteins named protein acyltransferases (PATs) with zinc-finger and aspartate–histidine–histidine–cysteine (DHHC) motifs [8, 9]. Multiple groups showed that members of the zDHHC family can both inhibit [10] and promote tumorigenesis by modulating S-palmitoylation of crucial proteins such as EGFR [11] and PD-L1 [12].

In our earlier work, we have identified Zinc Finger DHHC-Type Containing 1 (ZDHHC1) as a potential tumor suppressor that is hypermethylated in many cancers [13]. We further investigated the features of ZDHHC1 and found that ZDHHC1 suppressed tumor cell growth by activating p53 on many levels, most importantly by mediating p53 S-palmitoylation. Interestingly, p53 in turn induced *ZDHHC1* hypermethylation by recruiting DNMT3A to the p53 binding motif in the *ZDHHC1* promoter. These findings suggest a novel form of PTM for p53 that is critical in maintaining the activation of the p53 pathway, which is a part of an epigenetic regulatory loop between ZDHHC1 and p53, thus shedding light on the regulation of p53 pathway without the occurrence of genetic mutations and providing potential therapeutic targets for the battle against cancer.

## Results

### ZDHHC1 is suppressed in *TP53*^*WT*^ cancers and ectopic ZDHHC1 inhibits the proliferation of *TP53*^*WT*^ cancer cells

**Fig. 1:**
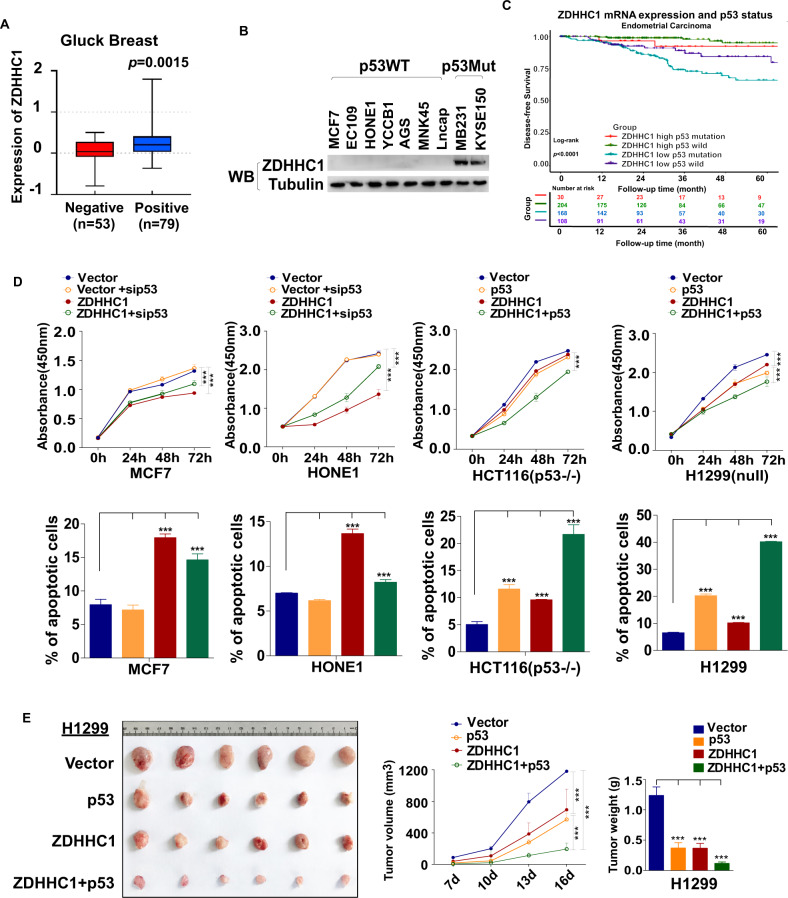
*ZDHHC1* expression and tumor-suppressing function correlate with p53 status. **A***ZDHHC1* mRNA expression in breast cancer patients [14] grouped by p53 IHC results. **B** ZDHHC1 protein expression in human cancer cell lines grouped by p53 mutation. **C** Disease-free survival of endometrial carcinoma patients according to *ZDHHC1* mRNA expression and p53 mutation status. See also Fig. S1B. **D** CCK8 and flow cytometric analyses of human cancer cell lines’ proliferation and apoptosis respectively. See also Fig. S2A–D. **E** Volume and weight of H1299 xenografts. See also Fig. S3A–B. Values represent mean ± SEM. ***p < 0.001.

Our previous work suggested that ZDHHC1 was silenced or downregulated in many cancer cell lines and tissues due to promoter DNA hypermethylation [13]. We’ve also noticed a correlation between ZDHHC1 expression and *TP53* mutation. To corroborate the notion, we first established that ZDHHC1 expression was quite ubiquitous in normal human tissues (Fig. S1A). In a cohort of 132 breast cancer samples [14], ZDHHC1 mRNA expression is positively correlated with p53 immunohistochemistry staining positivity, which is considered a sign of *TP53* mutation (Fig. 1A). Western blot showed ZDHHC1 expression patterns in human cancer cell lines being consistent with earlier RT-PCR results [13], which is mostly silent in *TP53*^*WT*^ cells but not *TP53*^*Mut*^ cells (Fig. 1B). Disease-free survival favors the groups of carcinoma patients with wild-type p53 and high ZDHHC1 expression while the group with mutated p53 and lower ZDHHC1 had the shortest disease-free survival (Fig. 1C; Fig. S1B). On the cellular level, ZDHHC1 inhibited tumor cell growth by inducing apoptosis when ectopically expressed in multiple human cancer cell lines [13]. However, this anti-tumor effect was only observed in cell lines without p53 mutations, which included HONE1 (nasopharynx) and MCF7 (breast). Knockdown of p53 in these cells effectively attenuated the effects of ZDHHC1 (Fig. 1D), suggesting ZDHHC1’s anti-tumor functions is noticeably dependent on p53^WT^. This notion was further substantiated by the in vitro growth assay of HCT116^*TP53-/-*^ and H1299 (ZDHHC1-positive, *TP53*^*null*^) cells. Overexpression of ZDHHC1 or p53 had a limited impact on the proliferation and apoptosis of the cells, but when ZDHHC1 and p53 were overexpressed simultaneously, a synergistic inhibitory effect can be seen (Fig. 1D; Fig. S2A–D). In a nude mouse xenograft tumor model, the synergy was confirmed in vivo as both p53 and ZDHHC1 impaired the growth of H1299 xenografts but the effect was significantly stronger when both were ectopically expressed (Fig. 1E; Fig. S3A; Fig. S3B). Collectively, these results suggest that ZDHHC1 is a p53-dependent tumor suppressor, and yet ZDHHC1 expression is suppressed in most p53^WT^ cancer cells.

### ZDHHC1 interacts with p53 and activates p53 signaling by promoting transcription and stabilizing p53 protein

**Fig. 2:**
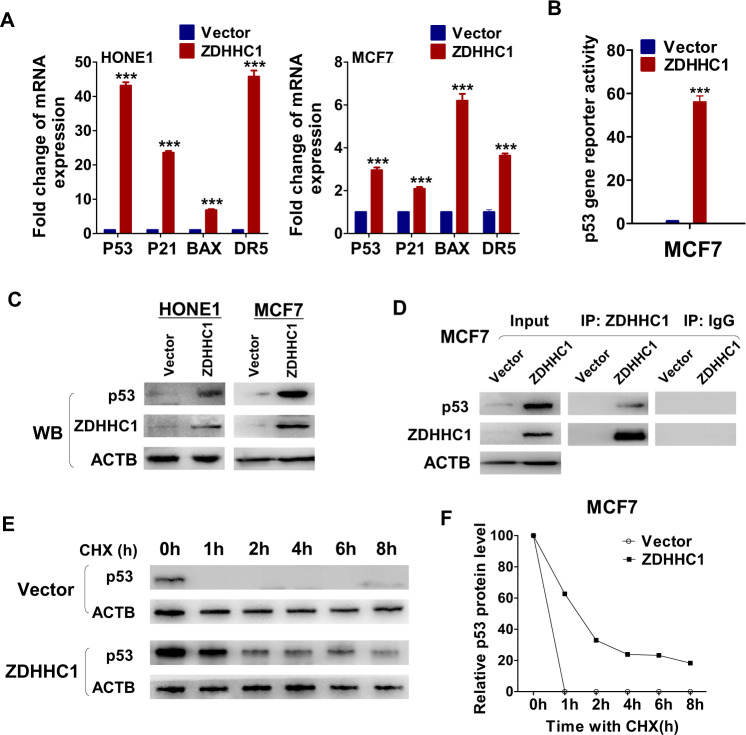
*ZDHHC1* activates p53 signaling through transcriptional and post-translational regulations. **A** mRNA expression of p53 target genes by qRT-PCR. **B** Transcriptional activity of *TP53* promoter in MCF7 cells. **C** Protein expression of p53 and ZDHHC1 by Western blot. **D** Co-IP analysis of ZDHHC1-p53 interaction in MCF7 cells. **E**, **F** p53 degradation rate analysis by Western blot and cycloheximide (CHX) treatment in MCF7 cells. Values represent mean ± SEM. ***p < 0.001.

To understand the connection between *ZDHHC1* and *TP53*, we first focused on the effect of ZDHHC1 on the p53 signaling pathway. When ZDHHC1 was ectopically expressed in HONE1 and MCF7 cells, classic targets of *P53* such as *P21*, *BAX*, and *DR5* were transcriptionally activated, along with p53 itself (Fig. 2A). ZDHHC1 has almost no effect in H1299 and very little effect in MB231 (*TP53*^*mut*^) on p53 target gene expression (Fig. S3C). The increased transcription of *TP53* was further confirmed by luciferase reporter assay in MCF7 (Fig. 2B). On the protein level, p53 expression also increased due to ectopic ZDHHC1 (Fig. 2C). Both ectopic and endogenous ZDHHC1 protein interacted with p53 protein in MCF7 and HCT116 cells respectively (Fig. 2D; Fig. S3D). Similar to many p53 agonists, ZDHHC1 extended the short half-life of wild-type p53 protein under protein synthesis inhibitor cycloheximide (CHX) treatment (Fig. 2E–F). These findings indicate that ZDHHC1 plays an important role in the p53 signaling pathway through multiple means of regulation, including the possibility of p53 being a substrate for the acyltransferase based on the Co-IP results.

### ZDHHC1 promotes p53 signaling through p53 palmitoylation

**Fig. 3:**
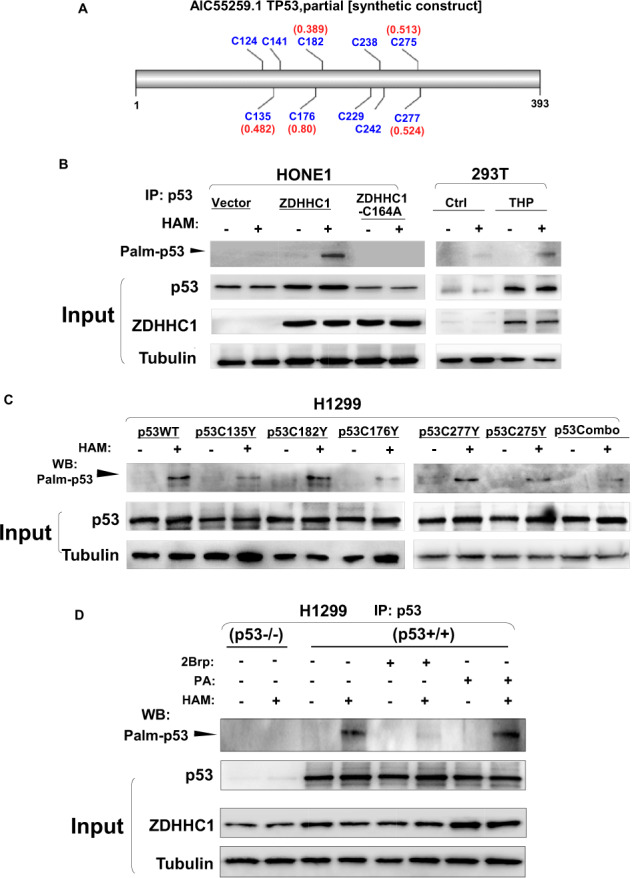
ZDHHC1 mediates p53 palmitoylation. **A** Predicted cysteine residues on p53 susceptible to S-palmitoylation. **B** IP-ABE analysis of p53 palmitoylation in HONE1 and in 293T cells were treated with pirarubicin (THP). **C** IP-ABE analysis of p53 palmitoylation in H1299 cells when ectopically expressing p53^WT^ or p53 harboring specific mutations. **D** IP-ABE analysis of p53 palmitoylation in H1299 cells when treated with palmitoylation inhibitor 2Brp or promoter PA. HAM hydroxylamine, 2Brp 2-bromopalmitate, PA Palmitic acid.

In a previous study, Cao and colleagues proposed that protein palmitoylation is required for p53-mediated DNA damage response, but no specific enzyme was identified [15]. Our data demonstrated a substantial correlation between ZDHHC1 and p53, including protein interaction. To verify if p53 is a target of ZDHHC1-mediated S-palmitoylation, we first utilized the palmitoylation site prediction tool CSS-PALM2.0 (http://www.csspalm.biocuckoo.org/). Among the potential cysteine residues within p53, C135, C176, C182, C275, and C277 have the highest scores (Fig. 3A). We then examined if the p53-ZDHHC1 interaction resulted in p53 palmitoylation. Co-IP coupled with ABE assay was performed [16] and indeed, palmitoylated p53 was detected in HONE1 cells, but only when ZDHHC1 was ectopically expressed (Fig. 3B). As the DHHC motif is essential for the catalytic activity of palmitoyl-transferases, a mutation (C164A) was introduced to the DHHC motif in ZDHHC1, which abolished p53 palmitoylation (Fig. 3B). Both p53 and ZDHHC1 protein expression elevated when 293 T cells were treated with pirarubicin (THP), along with p53 palmitoylation (Fig. 3B), suggesting the form of lipidation is involved in p53’s response to cellular stress. When the aforementioned p53 cysteine residues were respectively mutated and ectopically expressed in H1299 cells, the level of p53 palmitoylation decreased compared to the p53^WT^ group (Fig. 3C). This effect accumulated when all five forms of mutated p53 are ectopically expressed together (p53Combo). The increase in palmitoylated p53 was almost completely negated and slightly enhanced in the presence of palmitoylation inhibitor 2-bromopalmitate (2Brp) and palmitic acid (PA) respectively (Fig. 3D). Taken together, the results indicated that p53 is susceptible to ZDHHC1-mediated palmitoylation at certain cysteine residues, which expanded upon the findings of Cao’s group and the current understanding of p53 PTMs.

### ZDHHC1-mediated p53 palmitoylation is a prerequisite for p53 nuclear translocation

**Fig. 4:**
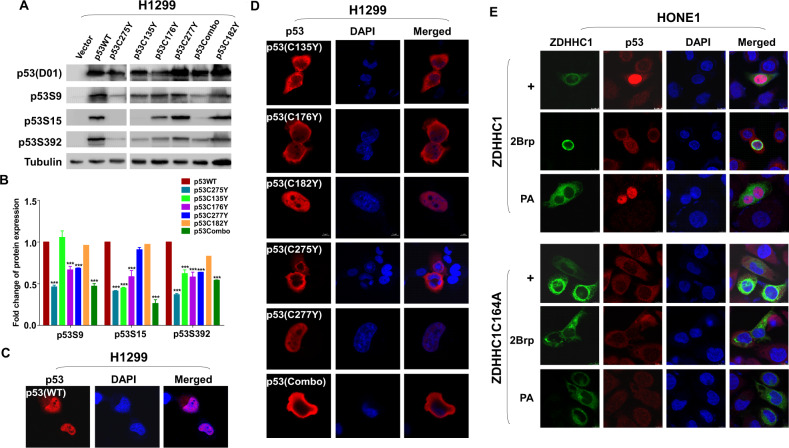
ZDHHC1 mediates p53 phosphorylation and nuclear translocation. **A** Western blot analysis of the correlation between site-specific p53 mutations and p53 phosphorylation. **B** Quantification of the Western blot results in Fig. 4A. **C** Subcellular localization of ectopic p53^WT^ in H1299 cells. **D** Subcellular localization of ectopic p53 with specific Cys substitutions in H1299 cells. **E** Subcellular localization of ZDHHC1 (top) or ZDHHC1-C164A (bottom) and p53 in HONE1 cells when treated with 2Brp or PA. Values represent mean ± SEM. *p < 0.05. ***p < 0.001. 2Brp 2-bromopalmitate, PA Palmitic acid.

**Fig. 5:**
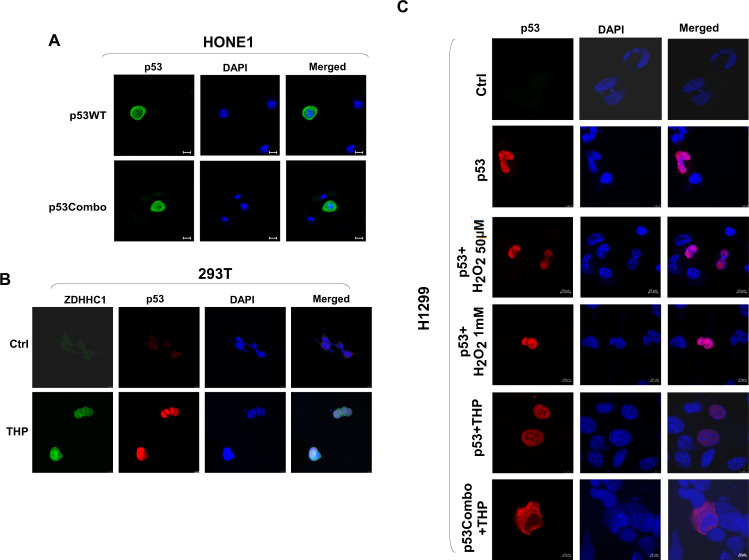
ZDHHC1 is necessary for p53 protein translocation from cytosol to nucleus. **A** Subcellular localization of p53^WT^ and p53Combo in HONE1 cells by immunofluorescent staining. **B** Subcellular localization of ZDHHC1 and p53 in 293 T cells after THP treatment by immunofluorescent staining. **C** Subcellular localization of ectopic p53 or p53Combo in H1299 cells after THP or H_2_O_2_ treatment by immunofluorescent staining. Pictures were photographed using confocal microscopy. THP: Pirarubicin.

Besides palmitoylation, one of the most consequential forms of protein modification for p53 is phosphorylation as it dictates the nuclear translocation of the transcription factor. We were interested to see if palmitoylation could influence the phosphorylation pattern or the subcellular localization of p53. We ectopically expressed the different forms of mutated p53 in H1299 cells. The phosphorylation of p53 at S9, S15, or S392 decreased in the single mutation groups and the p53Combo group compared with the p53^WT^ group (Fig. 4A, B), especially when C135, C176, and C275 were substituted. There was no observable change in other potential phosphorylation sites including S6, S20, S46, T18, and T81 (data not shown). Immunofluorescent staining showed that in H1299-p53^WT^ cells, p53 can accumulate in the nucleus (Fig. 4C). However, when the cysteine residues susceptible to palmitoylation were mutated into tyrosine, alanine, or serine residues, especially at the three sites that affected phosphorylation the most (C135, C176, and C275), nuclear translocation was significantly hindered (Fig. 4D; Fig. S4, right panel). Mutation of other cysteine residues did not stop p53 from entering the nucleus (Fig. S4, left panel) suggesting ZDHHC1-mediated p53 palmitoylation plays an important role in p53 phosphorylation and subcellular localization. Blocking palmitoylation with 2Brp also effectively suppressed the accumulation of p53 in the nucleus while PA had the opposite effect (Fig. 4E). Mutation of ZDHHC1 also retained p53 in the cytoplasm regardless of extrinsic palmitoylation regulators (Fig. 4E). Neither p53^WT^ nor p53Combo could abundantly accumulate in the nuclei of HONE1 cells when ZDHHC1 expression was not restored (Fig. 5A). These results further substantiate the necessity of ZDHHC1 in maintaining p53 nuclear translocation.

Next, we put cells under stress by treating cells with THP and obtained very similar results. The expression of ZDHHC1 and p53 in 293 T cells were triggered by THP treatment, and as suggested earlier, p53 mainly aggregated in the nucleus in the presence of ZDHHC1 (Fig. 5B). When the same treatment was applied to H1299 cells, ectopic p53^WT^ but not p53Combo was able to translocate to the nucleus (Fig. 5C).

**Fig. 6:**
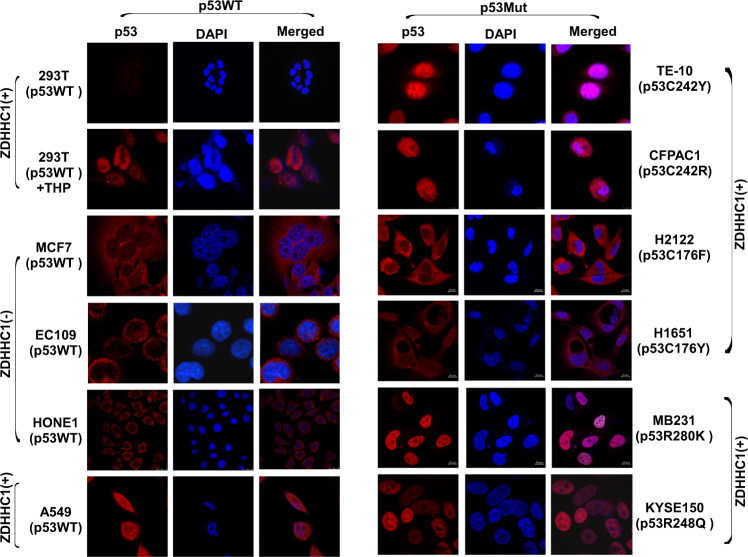
ZDHHC1 and p53 mutations determine p53 localization. **(Left)** Indirect immunofluorescent staining of p53^WT^ in 293 T (with or without THP treatment), MCF7, EC109, HONE1, and A549 cells. **(Right)** Indirect immunofluorescent staining of p53 in cells carrying varied mutant p53. TE10 (p53-C242Y), CFPAC1 (p53-C242R), H2122 (p53-C176F), H1651 (p53-C176Y), MB231 (p53-R280K), and KYSE150 (p53-R248Q) were enlisted as experimental subjects. Pictures were photographed using confocal microscopy. THP: Pirarubicin.

It was reported that p53 could be oxidized by hydrogen peroxide (H_2_O_2_) on zinc ion chelating amino acids H179, C238, C242, and C176 which alters the conformation and abolishes sequence-specific DNA binding of p53 [17]. However, treating cells with low or high concentrations of H_2_O_2_ did not affect the nuclear localization of p53 (Fig. 5C). p53 did not accumulate in the cell nucleus when ZDHHC1 expression was absent (MCF7, EC109, and HONE1) (Fig. 6, left panel) or when the C176 residue was mutated (H2122 and H1651) (Fig. 6, right panel). Mutations at other sites including other Cys residues (TE-10, CFPAC1, MB231, and KYSE150) did not stop p53 from entering the nuclei (Fig. 6, right panel). All of the above results indicate that ZDHHC1-mediated palmitoylation at specific cysteine residues was required for p53 nuclear translocation.

### ZDHHC1-mediated p53 palmitoylation is required for inhibiting tumor growth

**Fig. 7:**
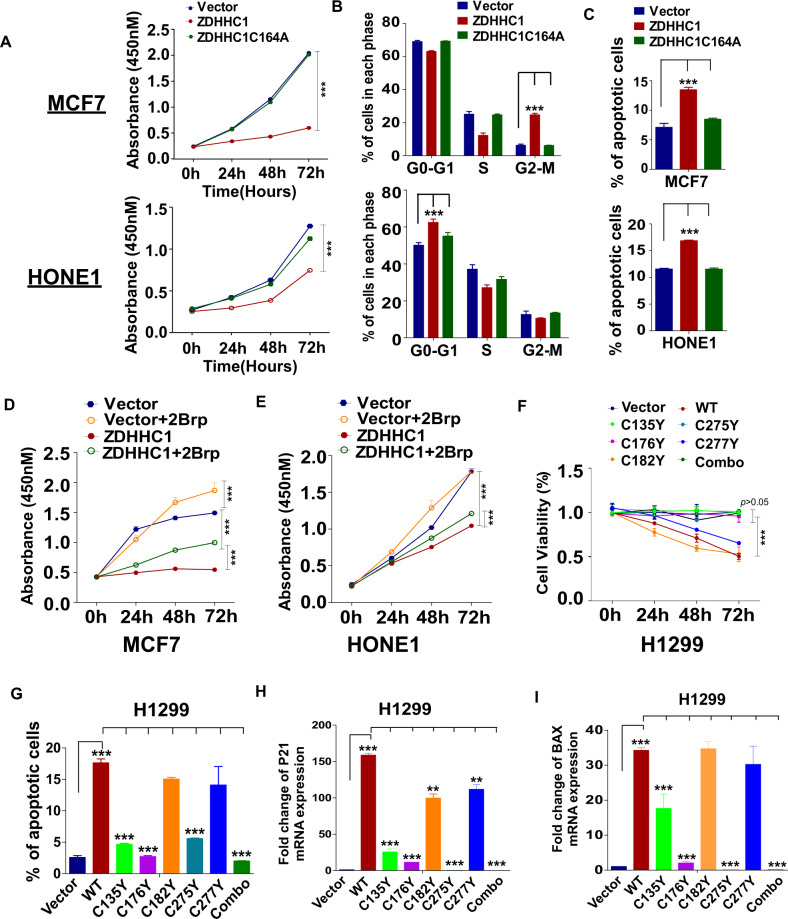
ZDHHC1-mediated p53 palmitoylation is required for suppressing tumor cell growth. **A** CCK8 proliferation assay of MCF7 and HONE1 cells. **B** Cell cycle analysis of MCF7 and HONE1 cells. See also Fig. S5A. **C** Flow cytometric analysis of apoptotic MCF7 and HONE1 cells expressing ZDHHC1 or ZDHHC1C164A. See also Fig. S5B. **D** CCK8 proliferation assay of MCF7 cells treated with 2Brp. **E** CCK8 proliferation assay of HONE1 cells treated with 2Brp. **F** CCK8 proliferation assay of H1299 cells ectopically expressing different variants of p53. **G** Flow cytometric analysis of apoptotic H1299 cells ectopically expressing different variants of p53. See also Fig. S6A–C. **H** mRNA expression of *P21* in H1299 cells by qRT-PCR. **I** mRNA expression of *BAX* in H1299 cells by qRT-PCR. Values represent mean ± SEM. **p < 0.01, ***p < 0.001.

We have demonstrated that p53 nuclear translocation is heavily dependent on ZDHHC1-mediated palmitoylation. So next we tried to determine if ZDHHC1-mediated p53 palmitoylation translates into phenotypic changes. Consistent with our previous observations, ZDHHC1 efficiently inhibited MCF7 and HONE1 proliferation (Fig. 7A), partly through inducing cell cycle arrest (Fig. 7B; Fig. S5A) and apoptosis (Fig. 7C; Fig. S5B). ZDHHC1-C164A lost such effect along with its functional DHHC motif which is necessary for the palmitoylation of substrates such as p53 (Fig. 7A–C; Fig. S5A–B). Inhibiting palmitoylation overall with 2Brp also reversed the inhibitory effect of ZDHHC1 on cell proliferation (Fig. 7D, E).

As we described earlier, p53 mutations at the C135, C176, and C275 residues had the strongest inhibition effect on ZDHHC1-mediated palmitoylation, phosphorylation, and nuclear translocation. Single mutated forms of ectopic p53 and p53Combo almost completely lost the ability to promote the expression of P21 and BAX, or suppress H1299 proliferation and induce apoptosis (Fig. 7F-I; Fig. S6A-C; Fig. S7). These results suggest that ZDHHC1-mediated p53 palmitoylation is a key mechanism for ZDHHC1’s tumor-suppressing functions.

### ZDHHC1 promoter hypermethylation is mediated by p53^WT^-recruited DNMT3A

**Fig. 8:**
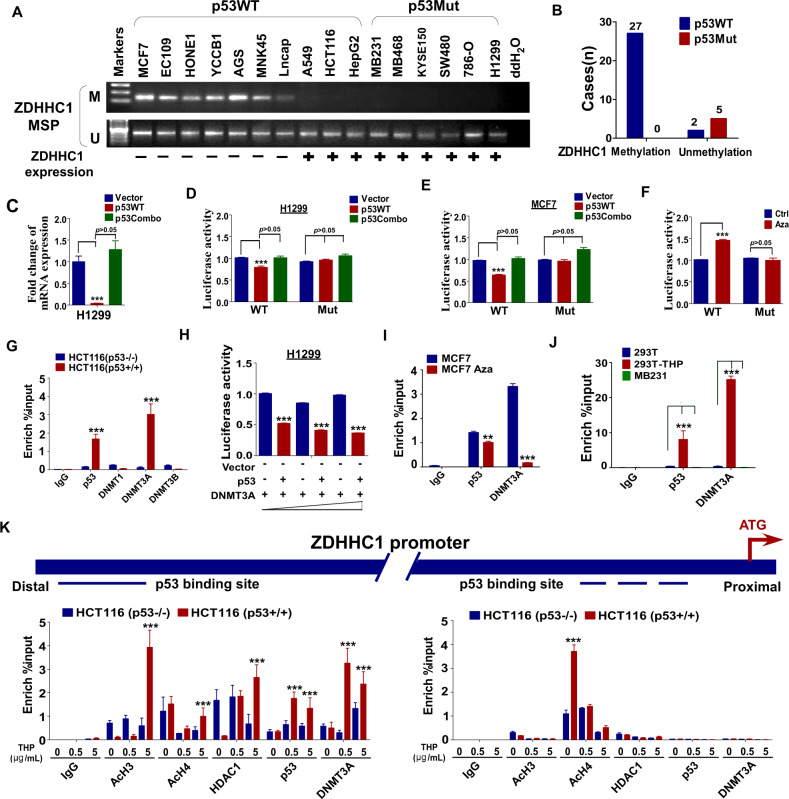
*ZDHHC1* methylation correlates with p53 mutation, and p53 recruits *DNMT3A* to *ZDHHC1* promoter to inhibit transcription. **A***ZDHHC1* promoter methylation in human cancer cell lines grouped by p53 mutation status. **B** Correlation between *ZDHHC1* promoter methylation and p53 mutation in 34 human breast cancer tissue samples. **C** mRNA expression of *ZDHHC1* in H1299 cells ectopically expressing p53^WT^ or p53Combo. **D** *ZDHHC1* promoter activity with or without mutating the p53 binding site in H1299 cells. **E** *ZDHHC1* promoter activity with or without mutating the p53 binding site in MCF7 cells. **F** *ZDHHC1* promoter activity with or without mutating the p53 binding site in MCF7 cells treated with Aza. **G** Enrichment of p53, DNMT1, DNMT3A, and DNMT3B near the p53 binding site on the *ZDHHC1* promoter by ChIP-qPCR. **H** *ZDHHC1* promoter activity in H1299 cells with ectopic p53 expression and different levels of ectopic DNMT3A expression. **I** Enrichment of p53 and DNMT3A near the p53 binding site on the *ZDHHC1* promoter before and after MCF7 cells were treated with Aza. **J** Enrichment of p53 and DNMT3A near the p53 binding site on the *ZDHHC1* promoter in 293 T cells treated with THP. MB231 cells were used as a control. **K** Enrichment of epigenetic regulators near the distal and proximal p53 binding sites on the *ZDHHC1* promoter. Values represent mean ± SEM. **p < 0.01, ***p < 0.001.

We previously found that the *ZDHHC1* promoter is hypermethylated in many cancers, causing the frequent lack of expression [13]. The silencing of *ZDHHC1* expression in the presence of promoter methylation and p53^WT^ respectively made us wonder if these events were correlated. Methylation-specific PCR (MSP) was conducted to examine the methylation status of *ZDHHC1* promoter in an array of human tumor cells. While most of the cell lines with wild-type *TP53* tested positive for methylation, none of the cell lines with mutated *TP53* did (Fig. 8A). When we separated human breast cancer samples by the state of p53 status, all 5 of the p53^mut^ samples tested negative for *ZDHHC1* promoter methylation. Most p53^WT^ samples were positive for methylation, only 2 out of 29 samples were unmethylated (Fig. 8B). We found frequent mutations in exon 7 of *TP53* in the unmethylated samples (Fig. S8). Exons 5, 6, and 8 also frequently harbor *TP53* mutations, but in these samples, no mutations were found in these regions (Fig. S8).

**Fig. 9:**
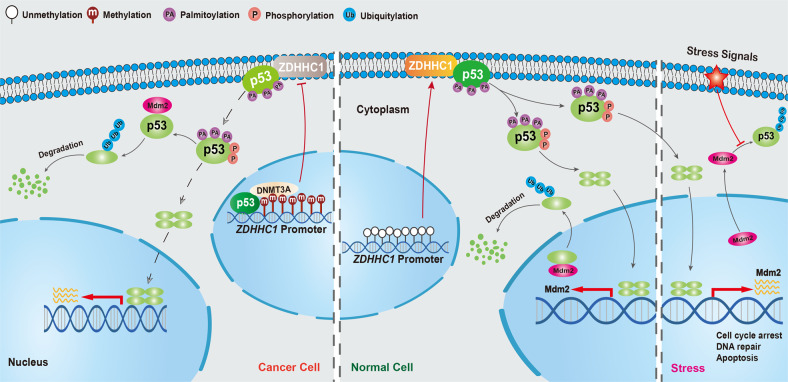
Graphic description of the epigenetic regulatory loop formed by *ZDHHC1*, p53, and DNMT3A. *ZDHHC1* interacts with p53 and mediates the attachment of palmitic acids to certain cysteine residues of the tumor suppressor, which is required for the subsequent phosphorylation and nuclear translocation that keeps cells in check when facing stress signals. This mechanism is shut off in many cancer cells due to the binding of the p53-DNMT3A complex to the CpG-rich region of *ZDHHC1* promoter and epigenetically silencing *ZDHHC1* expression, thus forming a negative regulatory loop that restricts p53 from entering the nucleus and releases the cell from p53’s control.

In H1299 cells, the mRNA expression of ZDHHC1 is significantly suppressed by ectopic p53^WT^ due to decreased transcriptional activity, but p53Combo failed to achieve similar effects (Fig. 8C, D). The same results were also observed in MCF7 cells (Fig. 8E). ZDHHC1 promoter activity was restored with DNA methylation inhibitor 5-aza-2’-deoxycytidine (Aza) (Fig. 8F), confirming our previous findings [13]. To identify the specific epigenetic regulators, we used ChIP-qPCR to analyze the promoter region of *ZDHHC1*. Results showed that p53 co-localized with DNMT3A at the potential p53 binding site, but not other DNA methyltransferases (Fig. 8G). DNMT3A inhibited *ZDHHC1* promoter activity in a dose-dependent manner (Fig. 8H). ChIP-qPCR was repeated after Aza demethylation treatment and the localized binding of DNMT3A significantly decreased (Fig. 8I). DNA damage by THP promoted the enrichment of both p53 and DNMT3A (Fig. 8J). We took a closer look at the promoter region of ZDHHC1. We found a potential p53 binding site next to a CpG island (Distal) and p53 binding sites located in close proximity to the ATG codon (Proximal). We designed ChIP-qPCR primers for both regions and p53/DNMT3A enrichment was only found at the distal site (Fig. 8K). On the p53 binding site (distal) near the CpG island, HDAC1 was also enriched under DNA damage (Fig. 8K). These results indicate that epigenetic regulation of *ZDHHC1* expression by p53 is not limited to DNA methylation, but also involve other mechanisms such as histone deacetylation. Taken together, these data point to a negative epigenetic feedback loop between p53 and *ZDHHC1* (Fig. 9) which provides clarification for previously reported findings and a possible mechanism for evasion of p53 tumor surveillance.

## Discussion

As the most prominent tumor suppressor gene, *TP53* had been under the spotlight for almost 4 decades [18]. However, the clinical application based on this celebrity gene is fairly limited due to the complexity of its regulatory network on multiple levels. Here we report a novel form of post-translational modification for p53 that is catalyzed by the palmitoyl S-acyltransferase (PAT) ZDHHC1 (zinc finger DHHC-type containing 1).

Post-translational modifications are common and crucial for p53, among which ubiquitination, phosphorylation, and acetylation are the most extensively studied [19]. Mdm2-p53 interaction has been well-established as the central node of the p53 pathway, and such interaction builds upon p53 ubiquitination [18]. When cells were introduced to certain forms of stress such as DNA damage, the Mdm2-p53 interaction moves in a direction in which p53 is phosphorylated and freed from Mdm2’s control. While phosphorylation-mediated stabilization and nuclear translocation as well as ubiquitination-mediated proteasomal degradation are major events for regulating p53 activity, there have been conflicting reports on the roles of other types of PTM [20].

Palmitoylation, or S-acylation, is a major type of reversible protein lipidation that is highly conserved in eukaryotic organisms [21]. The process of palmitoylation is mainly mediated by the zDHHC family, which links two of the most important macromolecules through covalent thioester bonds, altering the physical and chemical characteristics of proteins. Thousands of proteins could be susceptible to palmitoylation, implying the deep impact of such modification in biological and pathological settings [22]. Palmitoylation of p53 hasn’t been reported but two studies hinted at the possibility. In the first study, inhibiting palmitoylation with 2Brp effectively blocked mouse osteoblast differentiation, but not in *p53*^*-/-*^cells [23]. DNA damage-induced p53 activation in mouse embryonic fibroblasts could also be hindered by 2Brp [15]. The ZDHHC1-p53 protein interaction and the covalent attachment of palmitoyl groups on p53 cysteine residues may provide one plausible explanation, among many, for the effect of 2Brp on p53 activation.

The addition of lipids alters the protein’s affinity for water, therefore affecting the protein’s intracellular localization [24]. Our results indicate that palmitoylated Cys residues reduced the amount of substrate proteins in the cytoplasm and concentrated in the nucleus, yet there is no evidence showing palmitoylation affects p53’s affinity to nuclear membranes. It has been reported that importin alpha can be palmitoylated and bind to the plasma membrane, decreasing its level in the cytoplasm [25]. The opposite direction of transport between p53 and importin alpha could possibly be explained by the difference in the palmitoylation-phosphorylation interaction. Lipidation of p53 led to increased levels of phosphorylation at multiple sites while phosphorylation hindered importin α palmitoylation. Interestingly, importin alpha itself is a crucial regulator of protein nuclear transport [26]. Therefore, we speculate ZDHHC1-mediated palmitoylation enhanced p53’s affinity for the lipid bilayer that forms the nuclear envelope, thus creating a more friendly microenvironment for the nuclear import of p53, possibly with the involvement of importin alpha itself.

As our data suggested, C135, C176, and C275 out of the 5 candidate Cys residues are the most likely targets of ZDHHC1-mediated palmitoylation, yet these residues are also involved in the regulation of p53 activity through alternative mechanisms. C135 and C176 also enable the binding of p53 to NM23-H1 and STRAP respectively, resulting in the disassociation of p53-Mdm2 complex and p53 stabilization [27]. Oxidation of C176 by H_2_O_2_ was shown to inhibit the DNA binding ability [28]. But in our case, H_2_O_2_ didn’t seem to interfere with palmitoylation and nuclear transport. C275 mutation was found in lung cancer tissues [29] while it was the only 1 of 8 cysteine residues within p53 that affected the promoter binding affinity of Gadd45. Our results revealed that ZDHHC1 not only modulates the palmitoylation of p53 but also impacts the phosphorylation at multiple residues. The effect on p53 phosphorylation is likely one of the mechanisms by which ZDHHC1 promotes the transportation of p53 into the nucleus.

We have previously identified promoter DNA hypermethylation as a major cause of the silencing of *ZDHHC1* in tumors [13]. Now we have reason to believe that p53 plays a critical role in causing this epigenetic event, which completes the negative feedback loop. Our data showed that p53 can bind to the promoter of *ZDHHC1*, mostly next to a CpG island, and recruits the epigenetic writers DNMT3A and HDAC1 to the region to silence ZDHHC1 expression. In a few p53^WT^ cell lines (A549, HCT116, and HepG2), the promoter region of *ZDHHC1* was unmethylated, which could be explained by their unique genetic and/or epigenetic backgrounds that could affect the ZDHHC1 promoter methylation process. A similar trend can be seen in breast tumor tissue samples, each of its own heterogenous characteristics. 2 out of 29 p53 WT tissue samples tested in this study fall out of the box by being negative for ZDHHC1 promoter methylation. We propose DNMT3A to be the major epigenetic writer in this process, but other factors could also be at play. For example, HepG2 harbors a DNMT3B mutation [30] which may interfere with the de novo DNA methylation process. To a certain extent, this negative feedback loop could protect the tumor cells from the synergistic anti-tumor effect of p53 and ZDHHC1, and be one of the reasons why many tumors don’t bear *TP53* mutations yet still thrive.

From the clinical point of view, the dependent lethality of p53 and *ZDHHC1* translates into differences in prognosis. Many chemotherapy reagents eradicate tumor cells by inducing DNA damage and subsequent pathway activation. Through the negative feedback of p53 and *ZDHHC1*, there may be potential for predicting chemosensitivity by detecting the level of *ZDHHC1* promoter methylation, which can be easily achieved using both solid and liquid biopsy samples. The biomarker value of *ZDHHC1* will need further validation with more data on clinical samples, which we are collecting and will be thoroughly examined. Therapeutics that target palmitoylation is also still lacking due to insufficient knowledge on the detailed mechanisms. Contradicting evidence suggests that palmitoylation may need to be precisely targeted in different types of malignancies and in distinct genetic backgrounds [31].

Based on our results, we propose palmitoylation as a novel form of post-translational modification for p53, which is mediated by ZDHHC1 (Fig. 9). Palmitoylation is necessary for p53 nuclear trafficking and subsequent pathway activation. In turn, p53 regulates ZDHHC1 expression epigenetically by recruiting DNMT3A and HDAC1 to the promoter region. The regulatory feedback loop could help explain the development of p53-wildtype cancers and may be utilized for the advance of p53 targeted therapy.

## Materials and methods

### Cell lines, tumor samples, and normal tissues

Cell lines (HONE1, MCF7, A549, EC109, HCT116, HCT116^*TP53*-/-^, TE-10, CFPAC1, H2122, H1651, MB231, KYSE150, and H1299) were provided by ATCC (American Type Culture Collection, Manassas, VA, USA) or its collaborators. All cell lines were cultured in RPMI1640 (Gibco-BRL, Karlsruhe, Germany) media in general use and regularly tested for mycoplasma contamination. All cell lines were either recently purchased or recently authenticated by STR profiling. All tissues samples were collected from the First Affiliated Hospital of Chongqing Medical University, including all the primary tumor tissues and adjacent normal tissues. Certified pathologists verified that at least 70% of the cells in tumor samples are tumor cells. This research was authorized by the Institutional Ethics Committees of the First Affiliated Hospital of Chongqing Medical University (Approval notice: # 2016-61) and complied with the Declaration of Helsinki. Informed consent was obtained from all patients

### Reverse transcription-PCR and Quantitative real-time PCR

RNA was isolated from samples using Trizol according to the manufacturer’s introductions. DNA was extracted with QIAamp DNA mini kit (Qiagen, Hilden, Germany). In RT-PCR, the total volume of each reaction mixture was 10 µl with 2 µl of cDNA, and GAPDH was used as the inner control. The sequence of primers is presented in table S1. RT-PCR conducted using the Go-Taq (Promega, Madison, WI, USA) system under the condition of 32 cycles for samples, and 23 cycles for GAPDH. Quantitative real-time PCR (qRT-PCR) was conducted on the HT7500 System (Applied Biosystems, US) following the unit manual. The denaturing, annealing, and extension temperatures were set at 95, 60, and 72 °C, respectively.

### Bisulfite conversion and methylation-specific PCR (MSP)

Genomic DNA was obtained from cells and tissues. Bisulfite conversion of DNA samples was conducted as previously reported [32]. The primers used for MSP were listed in table S1. AmpliTaq-Gold DNA Polymerase (Applied Biosystems, US) was employed to amplify target DNA. After electrophoresis on 2% agarose gels, the results were photographed by a gel imaging system (Bio-RAD Gel Doc XR + , USA). MSP analysis was performed as previously described [33].

### Construction of plasmids and stable cell lines

Coding regions of target genes were inserted into the pEGFP-C1 framework plasmids. Recombinant plasmid had been verified by sequencing. After transfection, stable cell lines were selected using G418 (300 μg/ml for HONE1, 700 μg/ml for MCF7). For verification of ectopic gene expression, TRI reagent/protein extraction kit (Thermo Scientific, #23225) was used to perform total RNA/protein extraction. DNA contamination was cleaned using DNase (Deoxyribonuclease, Ambion, Austin, TX, USA). Moreover, we performed RT-PCR to amplify target genes and Western Blot was used to confirm the protein expression. All plasmids utilized in the study are listed in table S2.

### Indirect immunofluorescent staining

First, cells were seeded on glass coverslips and fixed with 4% paraformaldehyde in PBS. 0.1% Triton X-100 in PBS was then used for permeabilization. 1% bovine serum album in PBS was used for blocking non-specific conjugation at room temperature. Subsequently, the primary antibodies were applied for incubation at 4 °C overnight, followed by the incubation of the DyLight-conjugated anti-rabbit or anti-mouse secondary antibodies at room temperature for 1 h. Next, DAPI (4,6-diamidino-2- phenylindole) was used to counterstain the nucleus at room temperature for 5 min. Imagines were obtained under a fluorescence microscope.

### Tumor xenografts model in nude mice

5 × 10^6^ cells in 0.1 ml PBS were injected into the flanks of each female BALB/c nude mouse (*n* = 6, 4–6 weeks old). During the following 16 days, a vernier caliper was utilized to measure the diameter of the tumor every three days. Tumor volume was calculated using the following formula: volume = length × width^2 × 0.52. The xenografts were removed after the mice had been sacrificed and then weighed. The number of mice used in this study was chosen based on the experience from previous experiments under similar conditions. The standard deviation of xenograft size (main outcome) was considered. The number of mice was chosen to ensure adequate power and to detect biologically relevant differences. No randomization was used in the experiments in the animal studies. Our experiments were approved by the Animal Experiment Ethics Committee of Chongqing Medical University.

### Flow cytometry

Flow cytometric analysis of cellular apoptosis was conducted as previously described [13]. Briefly, cells were double-stained with Annexin V-fluorescein isothiocyanate (FITC; BD Biosciences, San Jose, CA) and PI according to the manufacturer’s protocol. Cells were then analyzed using FACSCalibur^TM^ (BD Biosciences, San Jose, CA). Data were analyzed using the CellQuest^TM^ software (BD Biosciences, San Jose, CA).

### Western blotting

Western blot was conducted as previously described [34]. Briefly, cells were lysed in the lysis buffer containing a protease inhibitor cocktail. The lysate was centrifuged at 4 °C and 1000 g for 10 min. The supernatant was harvested and quantified for sodium dodecyl sulfate–polyacrylamide gel electrophoresis (SDS-PAGE). The following primary antibodies were used in this study: anti-ZDHHC1 (#ab223042, Abcam), anti-p53 (DO-1) (#sc-126, Santa Cruz Biotechnology, Inc), or Phospho-p53 Antibody Sampler Kit (#9919, Cell Signaling Technology), while Tubulin (#sc-8035, Santa Cruz Biotechnology, Inc) and ACTB (#sc8432, Santa Cruz Biotechnology, Inc) were used as the internal control. The secondary antibody incubation was set for 1 h at 37 °C. Enhance Chemiluminescence detection kit (Amersham Pharmacia Biotech, Piscataway, NJ, USA) was used for the detection and analysis of protein expression.

### Luciferase reporter assay

During the construction of plasmids, the reporter was ligated in pGL3/Basic plasmid together with the target gene. PCR-amplified promoter regions and the reporter sequence were inserted into the pGL3/Basic plasmid using a seamless cloning kit (D7010S, Beyotime, Beijing, China). The plasmids were co-transfected with Renilla luciferase reporter pRL-TK (Promega) functioning as an internal control. Luciferase activity was measured after 48 h of transfection using a dual-luciferase reporter assay kit (Promega) following the manufacturer’s instructions. All experiments were performed in triplicate.

### Co-immunoprecipitation (Co-IP) assay

Cells were suspended in RIPA buffer and subsequently lysed via sonication. Protein quantitation was conducted using the BCA Protein Assay Kit (Pierce, Thermo Fisher Scientific, US) following the manufacturer’s manual. To pre-clear the cell lysate, A-sepharose magnetic beads were used. After incubation at 4 °C for 30 min, immunoprecipitating antibodies were then added into pre-cleared cell lysate. The immune complex was separated under the assistance of A-sepharose magnetic bead slurry. The beads were washed and the supernatant was used for immunoprecipitation (See Western blot methods).

### Co-IP-ABE assay

Co-IP coupled with ABE (Co-IP-Acyl-Biotin-Exchange) assay was performed as previously described [35]. Cultured cells are lysed, a target protein is then purified using a target-specific antibody, and immobilized on sepharose beads coated with protein G or A. The purified target protein is then treated with N-Ethylmaleimide (NEM) to irreversibly bind and block free thiol (-SH) groups along unmodified cysteines (C). The target protein is then subjected to treatment with hydroxylamine (HAM), resulting in specific cleavage of thioester bonds at palmitoylated cysteines and the unmasking of a free palmitoylated thiol group (-SH). Next, the target protein is treated with a thiol-reactive biotin molecule, biotin-BMCC, resulting in specific biotinylation of the palmitoylated cysteine. Finally, the biotinylated target protein is eluted and removed from the antibody and beads. The target protein with its palmitoylated cysteine(s) tagged with biotin is now suitable for SDS-PAGE, and western blotting with streptavidin to detect for palmitoylation of the purified protein.

### Chromatin immunoprecipitation (ChIP)-qPCR assay

ChIP was performed according to the manual of the SimpleChIP® Enzymatic Chromatin IP Kit (#9003, Cell Signaling Technology) [34]. Crosslinking of protein-DNA complexes was achieved with 1% formaldehyde before lysis (See Western blot methods). The lysed samples were sonicated to shear the chromatin and then incubated overnight with antibodies (Anti-p53: Cat. # 17-613, Millipore; Anti-acetyl-Histone H4: Cat. # 06-866, Millipore; Anti-acetyl-Histone H3: Cat. # 06-599, Millipore; Anti-Dnmt3a: ab2850, Abcam; Anti-Dnmt1: ab13537, Abcam; Anti-Dnmt3b: ab122932, Abcam) or normal IgG (#2729, Cell Signaling Technology) at 4 °C, followed by capture with protein A/G magnetic beads (#9006, Cell Signaling Technology) for 2 h. Then DNA was purified for the subsequent qPCR assays. ChIP-qPCR primers are listed in table S1.

### Bioinformatics and statistical analyses

NCBI Blast program http://www.ncbi.nlm.nih.gov/BLAST/ was used to analyze specific cDNA sequences. p53 transcription factor binding sites on the ZDHHC1 promoter were determined by the Jaspar database (Jaspar.genereg.net). All the data acquired were analyzed using SPSS VERSION.6 (Chicago, IL, USA). Two-tailed Student’s *t*-test, χ^2^ test, together with Fisher’s exact test was used according to the specific circumstance. The variance was similar between the groups that were statistically compared using a *t*-test. Statistical significance was recognized when *p* < 0.05.

## Supplementary information

SUPPLEMENTAL MATERIAL
