# Supplementary material for: Cancer cells escape p53’s tumor suppression through ablation of ZDHHC1-mediated p53 palmitoylation
Source: Oncogene. 2021 Jul 19;40(35):5416–26. doi: 10.1038/s41388-021-01949-5 (PMC8413129; doi:10.1038/s41388-021-01949-5)
Supplement: Supplementary file 1 — SUPPLEMENTAL MATERIAL [file 41388_2021_1949_MOESM1_ESM.docx]

**Supplementary tables**

**Table S1. List of PCR primers used in this study**

| **PCR** | **Primer** | **Sequence (5'-3')** | **Product size** | **PCR cycles** | **Annealing temperature** |
| --- | --- | --- | --- | --- | --- |
| **qRT-**  **PCR** | P21F | GTGGACAGCGAGCAGCTGAG | 104 bp | 40 | 55°C |
|  | P21R | AGTGGTGTCTCGGTGACAAAG |  |  |  |
|  | BAXF | TTTTGCTTCAGGGTTTCATC | 114 bp | 40 | 55°C |
|  | BAXR | GACACTCGCTCAGCTTCTTG |  |  |  |
|  | DR5F | CAGGACTATAGCACTCACTG | 160 bp | 40 | 55°C |
|  | DR5R | GGCACATCTCAGGAGAATCT |  |  |  |
| **MSP** | ZDHHC1m3 | TGGTCGTTGTGGGGCGTC | 112 bp |  | 60°C |
|  | ZDHHC1m4 | ACTCCAACAAACTAAAAAAACG |  |  |  |
|  | ZDHHC1u3 | GTTTGGTTGTTGTGGGGTGTT | 118 bp |  | 50°C |
|  | ZDHHC1u4 | CAACTCCAACAAACTAAAAAAACA |  |  |  |
| **ChIP-**  **qPCR** | ZDHHC1F1 | AGGAAGAATCTGGGAATGGGC | 134 bp |  |  |
|  | ZDHHC1R1 | TGACAACGGCGCGGGCTAC |  |  |  |
|  | ZDHHC1F2 | GAGGCTCTGAGCCTCAGGT | 92 bp |  |  |
|  | ZDHHC1R2 | CATCTCCCTCTGCCGGCT |  |  |  |
| **PCR** | p53E5-6F | GGGTTGCAGGAGGTGCTT | 585 bp | 35 | 55°C |
|  | p53E5-6R | TAAGCAGCAGGAGAAAGC |  |  |  |
|  | p53E7F | GCCACAGGTCTCCCCAAGGC | 275 bp | 35 | 55°C |
|  | p53E7R | GATGGGTAGTAGTATGGAAGAA |  |  |  |
|  | p53E8F | AGGGTGGTTGGGAGTAGATG | 319 bp | 35 | 55°C |
|  | p53E8R | GAGGCAAGGAAAGGTGATAAAA |  |  |  |

Note. qRT-PCR: Quantitative Reverse-transcription Polymerase Chain Reaction. MSP: Methylation-specific Polymerase Chain Reaction

**Table S2. List of plasmids used in this study**

| **Plasmid** | **Description** | **Source** |
| --- | --- | --- |
| pEGFPC1-ZDHHC1 | pEGFPC1 harboring ZDHHC1 | Le X et al，2020 |
| pcDNA-ZDHHC1 | pcDNA-SFiI-N4Flag harboring ZDHHC1 | This study |
| pEGFPC1-ZDHHC1-C164A | pEGFPC1 harboring ZDHHC1 with C164A mutation | Le X et al，2020 |
| pCMV-p53 | pCMV6-Entry harboring p53 | This study |
| p53C135Y | pCMV6-Entry harboring p53C135Y | This study |
| p53C176Y | pCMV6-Entry harboring p53C176Y | This study |
| p53C182Y | pCMV6-Entry harboring p53C182Y | This study |
| p53C275Y | pCMV6-Entry harboring p53C275Y | This study |
| p53C277Y | pCMV6-Entry harboring p53C277Y | This study |
| p53Combo | pCMV6-Entry harboring p53C135Y, C176Y, C182Y, C275Y, and C277Y | This study |
| pEGFP-p53 | pEGFPC1 harboring p53 | This study |
| pEGFP-p53C135Y | pEGFPC1 harboring p53C135Y | This study |
| pEGFP-p53C124A | pEGFPC1 harboring p53C124A | This study |
| pEGFP-p53C141A | pEGFPC1 harboring p53C141A | This study |
| pEGFP-p53C229A | pEGFPC1 harboring p53C229A | This study |
| pEGFP-p53C238A | pEGFPC1 harboring p53C238A | This study |
| pEGFP-p53C242A | pEGFPC1 harboring p53C242A | This study |
| pEGFP-p53C176Y | pEGFPC1 harboring p53C176Y | This study |
| pEGFP-p53C182Y | pEGFPC1 harboring p53C182Y | This study |
| pEGFP-p53C275Y | pEGFPC1 harboring p53C275Y | This study |
| pEGFP-p53C277Y | pEGFPC1 harboring p53C277Y | This study |
| pEGFP-p53C135A | pEGFPC1 harboring p53C135A | This study |
| pEGFP-p53C176A | pEGFPC1 harboring p53C176A | This study |
| pEGFP-p53C176S | pEGFPC1 harboring p53C176S | This study |
| pEGFP-p53C275A | pEGFPC1 harboring p53C275A | This study |
| pEGFP-p53Combo | pEGFPC1 harboring p53C135Y, C176Y, C182Y, C275Y, and C277Y | This study |
| pGL3-ZDHHC1 | pGL3-Basic harboring ZDHHC1 promoter (TSS+160-+400） | This study |
| pGL3-ZDHHC1-Mut | pGL3-Basic harboring ZDHHC1 promoter mutated | This study |
| pGL3-p53 | pGL3-Basic harboring p53 promoter | This study |
| sip53 | TP53 (Human) - 3 unique 27mer siRNA duplexes | This study |

**Supplementary figure legends**

**Fig. S1**

**(A)** Immunohistochemistry results demonstrating ZDHHC1’s distribution and expression in normal human breast, colon, esophagus, kidney and nasopharynx tissues. **(B)** Kaplan-Meier plots of correlation between disease-free survival and ZDHHC1 mRNA levels together with p53 status among Stomach Adenocarcinoma **(left)** and Hepatocellular Carcinoma **(right)** patients. Number of patients at risk from each group was also listed.

**Fig. S2**

Flow cytometry analysis of apoptosis. Cells were double-stained with propidium iodide and Annexin-V-FITC. **(A)** and **(B)** Apoptotic status of MCF7 and HONE1 cells transfected with Vector+siCtrl, Vector+sip53, ZDHHC1+siCtrl, and ZDHHC1+sip53 respectively. **(C)** and **(D)** Apoptotic status of HCT116 (p53 deficient) and H1299 cells transfected with Vector, p53, ZDHHC1 and ZDHHC1+p53 respectively.

**Fig. S3**

**(A)** Validation of ZDHHC1 and p53 expression in the xenografts. **(B)** HE Staining results **(top row)**, IHC staining results of ZDHHC1 **(middle row)** and TUNEL assay results **(bottom row)** were exhibited. Corresponding experiments were conducted with xenograft tumor tissue sections harvested from nude mice planted with MCF7 cells overexpressing Vector, p53, ZDHHC1, and ZDHHC1+p53 separately. **(C)** RT-qPCR analysis of mRNA expression of p53 and p53 target genes in MB231 and H1299 cells. **(D)** Co-IP analysis of endogenous ZDHHC1-p53 interaction in HCT116 cells.

**Fig. S4**

Indirect fluorescent staining of p53 in H1299 cell ectopically expressing p53 variants. Potential palmitoylation sites (C135, C275, and C176, **right panel**) and alternative cysteine residues (C124, C141, C229, C238, and C242, **left panel**) were substituted by alanine or serine residues.

**Fig. S5**

**(A)** Flow cytometry analysis of cell cycle distribution of MCF7 and HONE1 cells transfected with vector-, ZDHHC1, or ZDHHC1-C164A plasmids. respectively. **(B)** Flow cytometry analysis of cellular apoptosis of MCF7 and HONE1 cells transfected with vector-, ZDHHC1, or ZDHHC1-C164A plasmids.

**Fig. S6**

**(A)** Flow cytometry analysis of apoptosis. H1299 cells were transfected with vector plasmid or p53 variants, then double stained with propidium iodide and Annexin-V-FITC. **(B)** AO/EB staining of H1299 cells transfected with vector plasmid of p53 variants. **(C)** Representative AO/EB staining results. Values represent mean ± SEM. ***p < 0.001.

**Fig. S7**

CCK8 proliferation assay of H1299 cells ectopically expressing vector or mutated p53 (C135A, C176A, C176S, or C275A).

**Fig. S8**

Nucleotide base sequence of exon 5, 7, 6, and 8 of p53 under the condition of methylated or unmethylated ZDHHC1.

**Supplementary figures**

**Fig. S1**

**Fig. S2**

**Fig. S3**

**Fig. S4**

**Fig. S5**

**Fig. S6**

**Fig. S7**

**Fig. S8**
